# Computerized cognitive stimulation for people with dementia or with mild cognitive impairment: a bibliometric review

**DOI:** 10.1590/1980-57642021dn15-020003

**Published:** 2021

**Authors:** Sónia Rolland Sobral, Margarida Sobral

**Affiliations:** 1 Universidade Portucalense – Porto, Portugal.; 2 Psychogeriatrics Service, Hospital Magalhães Lemos – Porto, Portugal.; 3 Center for Health Technology and Services Research – Porto, Portugal.

**Keywords:** bibliometrics, cognition, computers, data analysis, dementia, bibliometria, estimulação cognitiva, computadores, análise de dados, demência

## Abstract

Many studies have found that non-pharmacological interventions, such as cognitive stimulation (CS), can benefit people with dementia (PWD) or with mild cognitive impairment (MCI). The use of the computerized cognitive stimulation (CCS) had proven to be an ally to those who want to detect and mitigate this disease.

**Objective:**

The purpose of this paper was to analyze the scientific production in CCS in PWD or with MCI in journals indexed in Clarivate Analytics’ Web of Science and Elsevier’s Scopus since 2000.

**Methods:**

Data collected from Web of Science and Scopus during 2000–2019.

**Results:**

The data show that dementia research is exponentially developing following the evolution of widespread use of computer science. As such, this article was of enormous importance doing a bibliometric analysis of what has been done in the area since the beginning of this century. The search terms identified 61 papers related to the use of computers applied to CS in PWD or MCI, and the International Journal of Geriatric Psychiatry and Journal of Alzheimer’s Disease had the largest number of publications. The most cited article was the Faucounau et colleagues. Major research’ countries are United Kingdom, Spain and United States.

**Conclusions:**

The findings in these papers were analysed to find recommendations for future work in this area. The CCS has been increasingly used as an intervention tool for PWD or MCI, and there still seems to be a possibility for evolution in good quality publications.

## RESUMO.

Muitos estudos têm demonstrado que as intervenções não farmacológicas, como a estimulação cognitiva (EC), podem beneficiar pessoas com demência (PCD) ou com declínio cognitivo ligeiro (DCL). O uso da estimulação cognitiva computadorizada (ECC) tem mostrado ser um meio para detetar e mitigar essa doença.

**Objetivo:**

O objetivo do presente artigo foi analisar a produção científica em ECC em PCD ou com DCL publicada em revistas indexadas na Web of Science da Clarivate Analytics e na Scopus da Elsevier desde 2000.

**Métodos:**

Os dados foram coletados na Web of Science e Scopus relativamente aos anos 2000–2019.

**Resultados:**

Os dados mostram que a pesquisa em demência está se desenvolvendo exponencialmente, acompanhando a evolução do uso generalizado da ciência da computação. Dessa forma, o estudo foi de enorme importância para uma análise bibliométrica do que tem sido feito na área desde o início deste século. Os termos de pesquisa identificaram 61 artigos relacionados ao uso de computadores aplicados à EC em PCD ou DCL, e ambos os periódicos *International Journal of Geriatric Psychiatry* e *Journal of Alzheimer’s Disease* tiveram o maior número de publicações. O artigo mais citado foi o de Faucounau et al. Os principais países de pesquisa foram Reino Unido, Espanha e Estados Unidos.

**Conclusões:**

Os resultados desses artigos foram analisados de forma a possibilitar encontrar recomendações para trabalhos futuros nessa área. A ECC tem sido cada vez mais utilizada como ferramenta nas intervenções para PCD e DCL, e ainda parece haver possibilidade de evolução em publicações de boa qualidade.

## INTRODUCTION

Dementia is a disorder characterized by deterioration of cognitive and functional abilities and several neuropsychiatry and behavioral symptoms.^1^ ^,^ ^2^ The most common dementia is Alzheimer’s disease (AD), which is a degenerative disease, what means that it becomes worse with time.^1^ The risk of dementia rises with increasing age, and this disease has a huge economic impact. Mild cognitive impairment (MCI) is characterized by an objective cognitive decline in one or more cognitive domains without any significant impairment in daily life activities.^3^ In fact, the MCI is the expected stage between the cognitive decline result of the normal aging and the more serious decline of dementia, and may increase the risk of dementia later developing caused by AD or other neurological conditions. Given the unprecedented personal, societal, and healthcare costs, it is not surprising that global efforts to develop and implement dementia risk reduction strategies are occurring.^4^ ^,^ ^5^

People with dementia (PWD) experience multiple symptoms that change over a period of years, and these symptoms reflect the degree of damage to nerve cells, wherein the pace at which symptoms of dementia advance from mild to moderate to severe differs from person to person.^1^ Though there is no cure for AD, there are some treatments available to ease symptoms and slow the disease progression.

Non-pharmacological interventions in combination with pharmacotherapies have been considered the best approach in the management of PWD^6^ ^–^ ^8^ and many studies have found that non-pharmacological interventions, such as cognitive stimulation (CS), can benefit PWD.^7^ ^,^ ^9^ ^–^ ^11^ There has been an interest in a variety of technological interventions that can treat symptoms of cognitive, behavioral and psychological impairment. CS is a non-pharmacological intervention to treat people with mild to moderate dementia.^12^ According to Piasek et al.,^13^ in the absence of a cure for dementia, there is a real need to develop user-centered technologies to enhance the well-being and quality of life of PWD.

The use of the computer, in its different types, has proven to be an ally to those who want to detect and mitigate this disease. Computerized cognitive training programs may be used as a practical and valuable tool in clinic to improve cognitive status.^14^ ^,^ ^15^ García-Casal et al.^16^ found that the computer-based cognitive interventions had moderate effects in cognition. However, they led to superior results compared to non-computer-based interventions in cognition.

Dementia and non-pharmacological interventions research, such as computerized cognitive stimulation (CCS), is exponentially developing following the evolution of widespread use of computer. As such, this article intended to be of enormous importance doing a bibliometric analysis about what has been done in the area since the beginning of this century. The motivation for this research was based on our desire to know and clarify the impact of non-pharmacological interventions using a computer and its advantages over the use of traditional methods of CS and to know what has been published in this area. This review is different from all other existing reviews, as it managed to cover a wide range of articles, with key information to understand the impact of the use of technologies in the areas of non-pharmacological interventions. The purpose of this paper was to analyze the scientific production in CCS and PWD or people with MCI in journals indexed in Clarivate Analytics’ Web of Science and Elsevier’s Scopus.

This document is divided into several sections. First, the research questions are described, followed by the methodology and the results. Then, a summary of the most cited articles is made, as well as an analysis of all documents in the database, ending with a discussion of the results obtained and the conclusions, including a suggested future work.

## THE RESEARCH QUESTIONS

The question, along with the purpose of the review, the intended deliverables and the intended audience, determines how the data are identified, collected and presented.^17^ There are several questions that we want to answer in this paper:

- Q1: When were the articles published?
- Q2: Where were the articles published?
- Q3: What is the focus of the articles? Has it evolved over the years?
- Q4: Who publishes on the subject? Where do researchers who are interested in PWD work? What country do they live in?
- Q5: What are the most cited articles?

## METHODS

The term “bibliometrics” was first used in 1969 by Alan Pritchard, hoping that it would be used explicitly in all studies which seek to quantify the processes of written communication and would quickly gain acceptance in the field of information science.^18^ Moed mentioned the potential of this type of study that reveals the enormous potential of quantitative, bibliometric analyses of the scholarly literature for a deeper understanding of scholarly activity and performance, and highlights their policy relevance.^19^ In scientific research, it is important to get a comprehensive perspective of research already being conducted concerning a relevant subject matter^20^ and a bibliometric analysis profile on the research trajectory and dynamics of the research activities across the globe.^21^ This is a bibliometric study that systematically analyses the literature using two at Elsevier’s Scopus (Scopus) and Clarivate Analytics’ Web of Science (WoS) databases. This paper conducts a bibliometric analysis of international papers that we expect to provide a useful reference for future research.

The research strategy was designed by the authors according to the literature review previously prepared. In this case, the terms that are identified as relevant to the present study were defined, the time frame (from 2000 to 2019), and the type of publication identified as relevant to the research.

The Scopus search strategy was:

- TITLE-ABS-KEY (“cognitive stimulation” AND comput* AND “dementia”).
- DocType: Article OR Review OR Conference Paper.
- PUBYEAR: >1999 AND <2020.

The WoS search strategy was:

- TS=(“cognitive stimulation” AND comput* AND “dementia”).
- Document Type = (ARTICLE OR MEETING OR REVIEW).
- PY=(2000–2019).

The eligible papers were required to: (a) include participants with a diagnosis of dementia or MCI using a validated cognitive screening measure; (b) examine the effects of CCS; (c) include case series, control group, randomized or non randomized design.

## RESULTS

**Figure 1:**
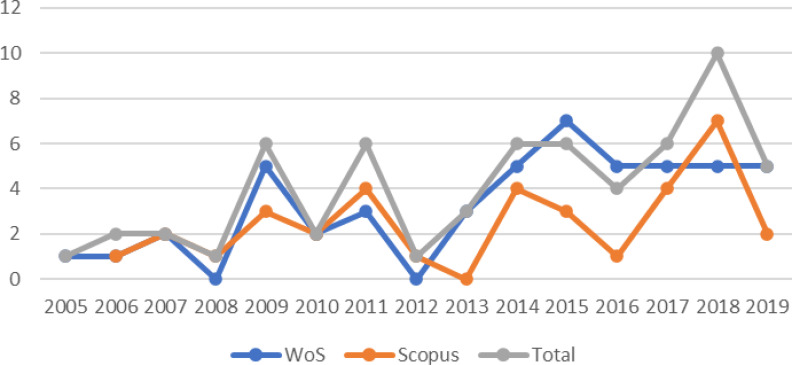
Evolution of published papers.

A set of 48 published papers were collected from WoS and 35 from Scopus. The search returned a total of 61 articles and reviews after discounting the duplicate results. Thus, this bibliometric study analyzed the literature using 17 articles from Scopus,^13^ ^,^ ^22^ ^–^ ^27^ 27 from WoS,^12^ ^,^ ^16^ ^,^ ^28^ ^–^ ^34^ and 17 articles indexed in both databases.^35^ ^–^ ^44^ In the articles studied, the trials included most were unregistered, parallel-group or single-site randomised controlled trials. The first article was published in 2005 (Figure 1). The average number of articles is 4 per year. The year with the greatest number of articles is 2018.^10^ We can observe in Figure 1 that both curves of publications have an increasing trend until 2018 in both datasets, due to the progress of scientific literature in this field of research. We noted that in the last year there has been a decrease in published papers.

There are 50 sources of publications: only two journals have three publications (*International Journal of Geriatric Psychiatry* and *Journal of Alzheimer’s Disease*) and seven others have two publications (one of these is ACM International Conference Proceeding Series) (Table 1). Most of these journals are from the first quartile, three are from United Kingdom and three from United States.

**Table 1:** Journals/conference information’s

Journal/Conference	#	Country	SJR 2018	IF 2018	WoS Subject (category)	Quartil	H Index
International Journal of Geriatric Psychiatry	3	United Kingdom	1.41	3.141	Medicine (Geriatrics and Gerontology; Psychiatry and Mental Health)	Q1	116
Journal of Alzheimer’s Disease	3	Netherlands		3.517	Neurosciences (Neuroscience & Behavior; Neurosciences & Behavior)		
ACM International Conference Proceeding Series	2	United States	0.17				98
Aging and Mental Health	2	United Kingdom	1.23	2.956	Medicine (Geriatrics and Gerontology; Psychiatry and Mental Health) Nursing (Gerontology; Psychiatric Mental Health)	Q1	74
Alzheimer’s and Dementia: Translational Research and Clinical Interventions	2	United States	1.5		Medicine (Neurology (clinical); Psychiatry and Mental Health)	Q1	13
Clinical Interventions in Aging	2	New Zealand	1	2.585	Medicine (Geriatrics and Gerontology) Medicine (miscellaneous)	Q2; Q1	59
Cochrane Database of Systematic Reviews	2	United States	1.61	7,755	Medicine (Medicine (miscellaneous); Pharmacology (medical))	Q1	244
Journal of the American Geriatrics Society	2	United Kingdom	2.13	4.113	Medicine Geriatrics and Gerontology	Q1	208
Lecture Notes of the Institute for Computer Sciences, Social-Informatics and Telecommunications Engineering	2	Germany	0.15		Computer Science (Computer Networks and Communications)	Q4	36

SJR: Scientific Journal Rankings; IF: impact factor.

Most articles are written by three authors (21%), 15% are written by five authors and 10% by two authors.

There are 320 authors. Only three of these 320 wrote three articles. The authors with three articles were Hiroko Hayama Dodge from the University of Michigan, (Ann Arbor, United States), Hermine Lenoir from the Université Paris Descartes (Paris, France) and Victoria Meza-Kubo from the Universidad Autónoma de Baja California (Ensenada, Mexico).

We found 425 keywords. The 20 most common keyword are “Dementia”, “aged”, “Cognitive stimulation”, “Article”, “Mild cognitive impairment”, “human”, “quality of life”, “Cognition”, “controlled study”, “Alzheimer disease”, “caregiver”, “cognitive defect”, “cognitive therapy”, “Neurodegenerative diseases”, “adult”, “aging”, “Alzheimer’s disease”, “Cognitive stimulation therapy”, “Cognitive stimulations” and “technology”.

**Figure 2:**
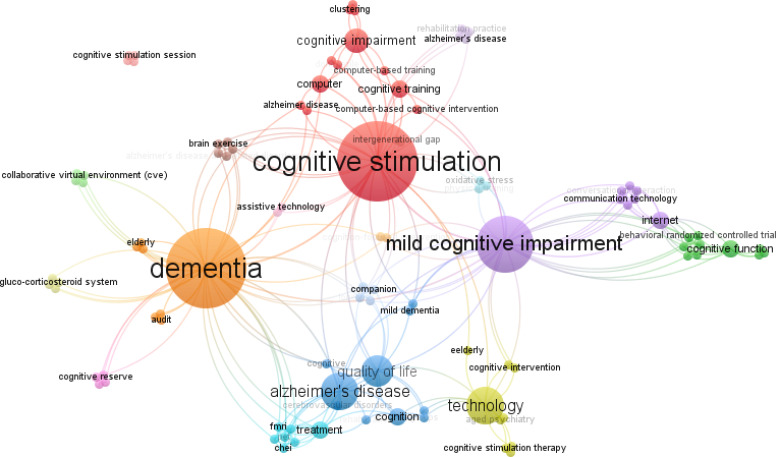
Network visualization, keywords.

In Figure 2, we can see the network visualization of the keywords, using VOSviewer - Visualizing scientific landscapes. We found three clusters:

- (C1) Alzheimer’s disease, cognition, quality of life, technology and treatment.
- (C2) Cognitive impairment, cognitive stimulation, cognitive training and computer.
- (C3) Cognitive function, internet and mild cognitive impairment.

Table 2 shows the most frequent keywords for different periods: until 2009, 2009–2014 and 2014–2019.

**Table 2:** Most frequent keywords for different periods.

Human	Dementia	Dementia
Alzheimer Disease	Aged	Human
Article	Human	Aged
Dementia	Cognitive Stimulation	Cognition
Donepezil	Female	Cognitive Stimulation
Humans	Humans	Cognitive Stimulations
Male	Male	Female
Single Photon Emission Computer Tomography	Mild Cognitive Impairment	Male
Adult	Alzheimer’s Disease	Mild Cognitive Impairment
Aged	Neurodegenerative Diseases	Article
Aged, 80 And Over	Quality Of Life	Cognitive Therapy
Alzheimer’s Disease	Aged, 80 And Over	Controlled Study
Cognition	Aging	Humans
Cognitive Defect	Article	Neurodegenerative Diseases
Cognitive Therapy	Cognition Disorders	Very Elderly
Female	Controlled Study	Aged, 80 And Over
Galantamine	Treatment Outcome	Cognitive Defect
Major Clinical Study	Alzheimer Disease	Computer Assisted Therapy
Neuroimaging	Cognition	Human Computer Interaction
Nuclear Magnetic Resonance Imaging	Cognitive Defect	Major Clinical Study
Positron Emission Tomography	Cognitive Impairment	Quality Of Life
Priority Journal	Cognitive Therapy	Caregiver
Risk Factors	Cognitive Training	Cognitive Stimulation Therapy
	Computer Science	Mild Cognitive Impairments
	Health Care	Patient Treatment
	Internet	Pilot Study
	Physical Activity	Priority Journal
	Review	Psychology
	Technology	Technology
	Ubiquitous Computing	
	Very Elderly	

The authors are from 20 different countries. The countries with the largest number of articles are: United Kingdom (15%), Spain (12%) and United States (10%).

The network of co-authorship countries has high density and a small number of clusters suggesting being centered on some countries. There are only two clusters with more than two countries:

- (C1) France, Greece, Ireland, Italy, New Zealand and South Korea.
- (C2) Canada, Netherlands, and United Kingdom.

**Figure 3:**
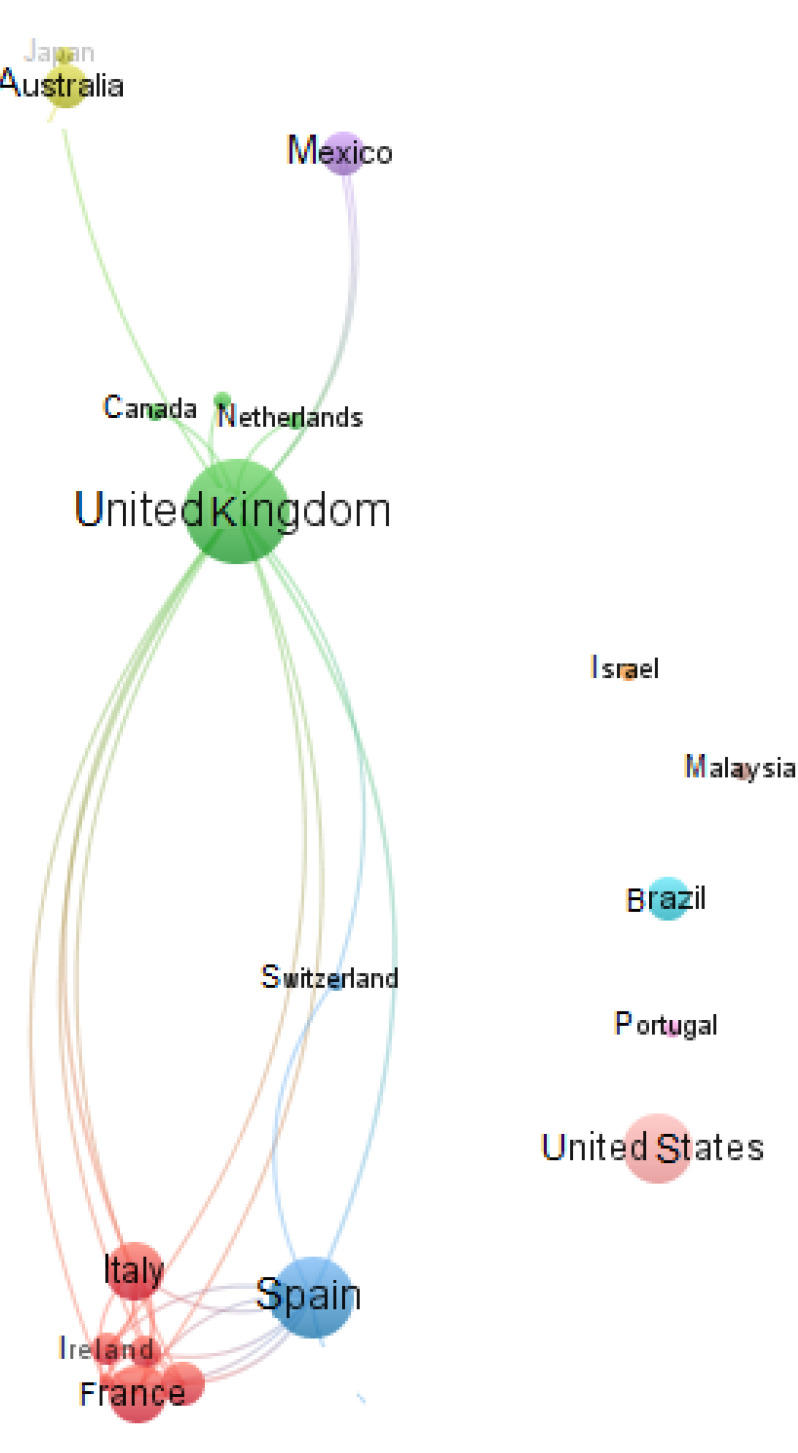
Network visualization, countries.

The development of the co-authorship country research collaboration in CCS and PWD is presented in the Figure 3 distributed by degree of centrality, using VOSviewer. It is a centered network around United Kingdom, Spain and United States, France and Italy, which have the highest number of links with other countries, co-authoring several articles and the biggest importance in the development of the field.

We find that there are 93 organizations: 12 of these organizations have more than 2 articles. The most productive organizations by the number of the articles in CCS and PWD for the all period (1999–2020) includes the Consiglio Nazionale delle Ricerche, Aristotle University of Thessaloniki, Ewha Womans University, Hospital Provincial de Zamora, OHSU School of Medicine, Oregon Health and Science University, Portland VA Medical Center, Universidad Autónoma de Baja California, Universidad de Salamanca, University of Hull, University of Melbourne and University of Michigan - Ann Arbor.

Because we didn’t use the language exclusion criteria, we can now see that English is used in 89% of the articles. The other languages are Spanish (6%), French (3%) and Korean (3%).

The affiliation of the most cited documents’ author is AP-HP Assistance Publique – Hopitaux de Paris (Paris, France), followed by Université Paris Descartes (Paris, France). The next table (Table 3) list the most cited author affiliation.

**Table 3:** Most cited documents’ author affiliation.

	Author	Affiliation
1	Faucounau^36^	AP-HP Assistance Publique - Hopitaux de Paris, Paris, France
	Wu^36^	Université Paris Descartes, Paris, France
	Boulay^36^	Université Paris Descartes, Paris, France
	Rotrou^36^	Université Paris Descartes, Paris, France
	Rigaud^36^	Université Paris Descartes, Paris, France
2	Viola^38^	Department and Institute of Psychiatry, Universidade de Sao Paulo, SP, Brazil
	Nunes^38^	Department and Institute of Psychiatry, Universidade de Sao Paulo, SP, Brazil
	Yassuda^38^	School of Arts, Sciences and Humanities, Universidade de São Paulo, SP, Brazil
	Aprahamian^38^	Department and Institute of Psychiatry, Universidade de Sao Paulo, SP, Brazil
	Santos^38^	Hospital da Faculdade de Medicina da Universidade de São Paulo, SP, Brazil
	Santos^38^	Department and Institute of Psychiatry, Universidade de Sao Paulo, SP, Brazil
	Brum^38^	Department and Institute of Psychiatry, Universidade de Sao Paulo, SP, Brazil
	Borges^38^	Department and Institute of Psychiatry, Universidade de Sao Paulo, SP, Brazil
	Oliveira^38^	Department and Institute of Psychiatry, Universidade de Sao Paulo, SP, Brazil
	Chaves^38^	Department and Institute of Psychiatry, Universidade de Sao Paulo, SP, Brazil
	Ciasca^38^	Department and Institute of Psychiatry, Universidade de Sao Paulo, SP, Brazil
	Ferreira^38^	Department and Institute of Psychiatry, Universidade de Sao Paulo, SP, Brazil
	de Paula^38^	Department and Institute of Psychiatry, Universidade de Sao Paulo, SP, Brazil
	Takeda^38^	Department and Institute of Psychiatry, Universidade de São Paulo, SP, Brazil
	Mirandez^38^	Department and Institute of Psychiatry, Universidade de São Paulo, SP, Brazil
	Watari^38^	Department and Institute of Psychiatry, Universidade de São Paulo, SP, Brazil
	Falcão^38^	School of Arts, Sciences and Humanities, Universidade de São Paulo, SP, Brazil
	Cachioni^38^	School of Arts, Sciences and Humanities, Universidade de São Paulo, SP, Brazil
	Forlenza^38^	Department and Institute of Psychiatry, Universidade de SP, SP, Brazil
3	García-Casal^16^	Universidad de Salamanca, Salamanca, Spain
	Loizeau^16^	Center for Gerontology, University of Zurich, Zurich, Switzerland
	Csipke^16^	University College London, Institute of Mental Health, London, UK
	Franco-Martín^16^	Hospital Universitario Río Hortega, Valladolid, Spain
	Perea-Bartolomé^16^	Universidad de Salamanca, Salamanca, Spain
	Orrell^16^	University of Nottingham, Nottingham, United Kingdom
4	González-Palau^40^	Fundación Intras, Valladolid, Spain
	Franco^40^	Hospital Zamora, Zamora, Spain
	Bamidis^40^	Greek Aerospace Medical Association and Space Research, Greece
	Losada^40^	Fundación Intras, Valladolid, Spain
	Parra^40^	Fundación Intras, Valladolid, Spain
	Papageorgiou^40^	University of Athens Medical School, Athens, Greece
	Vivas^40^	The University of Sheffield International Faculty, Sheffield, Greece
5	Venneri^57^	University of Hull, Hull, United Kingdom
6	Eckroth-Bucher^35^	Bloomsburg University, Bloomsburg, United States
	Siberski^35^	College Misericordia, Dallas, United States
7	Breton^22^	Universidad de Deusto, Bilbao, Spain
	Zapirain^22^	Universidad de Deusto, Bilbao, Spain
	Zorrilla^22^	Universidad de Deusto, Bilbao, Spain
8	Dodge^24^	University of Michigan, Ann Arbor, Ann Arbor, United States
	Zhu^24^	University of Michigan, Ann Arbor, Ann Arbor, United States
	Mattek^24^	Oregon Health and Science University, Portland, United States
	Bowman^24^	Oregon Health and Science University, Portland, United States
	Ybarra^24^	University of Michigan, Ann Arbor, Ann Arbor, United States
	Wild^24^	Oregon Health and Science University, Portland, United States
	Loewenstein^24^	University of Florida, Gainesville, United States
	Kaye^24^	Oregon Health and Science University, Portland, United States
9	Westphal^37^	University of Melbourne, Parkville, Australia
	Dingjan^37^	University of Melbourne, Parkville, Australia
	Attoe^37^	University of Melbourne, Parkville, Australia

According to Chen,^45^ the most cited papers give historical perspective on scientific progress and reveal recognition of scientific advancement. As usual in the literature, older papers receive more citations than recent one, given the time length of knowledge diffusion. Curiously, the majority of top cited articles belong to a more recent period, and the most cited paper was published in 2010.

The most cited article, with 50 citations, was the Faucounau et al.^36^ with the title “Cognitive intervention programmes on patients affected by mild cognitive impairment: a promising intervention tool for MCI?”. In this article, they found that though both traditional and computer-based cognitive intervention programs seem to be effective, the computer-based ones present more advantages, namely, the teams that work with patients with cognitive impairment could individualize the program tailored to the patient’s neuropsychological pattern and needs; the computer-based cognitive intervention programs allow the user to make an immediate objective comparison with data collected earlier and also help in setting up a systematic training plan by providing instant value-free feedback and enable this tool offer a possibility of a widescale dissemination. In a paper with 44 citations, Viola et al.^38^ reported that they implemented a multidisciplinary rehabilitation program on cognition, quality of life, and neuropsychiatry symptoms in patients with mild AD. The group sessions were provided by a multiprofessional team and included computer-assisted cognitive stimulation, memory training, expressive activities, physiotherapy, and physical training. They found that this multimodal rehabilitation program was associated with cognitive stability and significant improvements in the quality of life for Alzheimer’s patients. Recently, in 2017, in a paper with 33 citations, García-Casal et al.^16^ considered that computer-based cognitive interventions have moderate effects in cognition in PWD. However, they led to superior results compared to non-computer-based interventions in cognition. Another paper (with 27 citations), developed by Eckroth-Bucher and Siberski,^35^ found that blending computer-based with traditional cognitive stimulation activities showed promise in preserving cognitive function in older people. In 2014, in a paper, currently, with 33 citations, González-Palau et al.^40^ presented the results of a study using the newly integrated platform (The Long Lasting Memories program) which combined cognitive exercises with physical activity within the context of advanced technologies. This study indicated this program was a promising solution for older adults with and without cognitive impairment, maintaining their well-being with few professional and technical requirements. Breton et al.^22^ reported a tool focuses on therapeutic aspects of both cognitive and physical stimulation of the older people, that is, it improves the memory by performing mental activities and physical exercise at the same time. Preliminary tests have shown an increase in the users’ motivation while using the tool forgetting that it focuses on the CS. Using another approach to the use of CCS and PWD, Dodge et al.^24^ examined the feasibility of a randomized controlled trial to assess conversation-based cognitive stimulation through personal computers, webcams, and a user-friendly interactive Internet interface and they found that daily conversations by way of user-friendly Internet communication programs demonstrated high adherence. They concluded that the increasing daily social contacts through communication technologies could offer cost-effective home-based prevention methods. In Table 3, we can also see the reference to the paper by Westphal et al.^37^ with 14 citations. They identified and reviewed the latest research in the use of low and high technology in the areas of mood disorders, psychosis, normal ageing, mild cognitive impairment and dementia, and they found that research in the use of low and high technology in late-life mental disorders continued to evolve in its scope and innovation. These authors also considered that to make progress in accessibility and acceptability of technology, involvement of stakeholders and users in the design and application, as well as the examination of cost-effectiveness and robust methodologically designed studies, is necessary.

The Table 4 lists the affiliations of the authors of the most cited articles. Some of the most productive institutions involved in CCS and PWD are Department and Institute of Psychiatry, Universidade de São Paulo, São Paulo, Brazil and Université Paris Descartes, Paris, France.

**Table 4:** Affiliation author, most cited articles.

Affiliation	n
Department and Institute of Psychiatry, Universidade de São Paulo, São Paulo, Brazil	15
Universite Paris Descartes, Paris, France	4
Fundación Intras, Valladolid, Spain	3
Oregon Health and Science University, Portland, United States	3
School of Arts, Sciences and Humanities, Universidade de São Paulo, São Paulo, Brazil	3
Universidad de Deusto, Bilbao, Spain	3
University of Melbourne, Parkville, Australia	3
University of Michigan, Ann Arbor, Ann Arbor, United States	3
Universidad de Salamanca, Salamanca, Spain	2
AP-HP Assistance Publique - Hopitaux de Paris, Paris, France	1
Bloomsburg University, Bloomsburg, United States	1
Center for Gerontology, University of Zurich, Zurich, Switzerland	1
Hospital das Clínicas da Faculdade de Medicina da Universidade de São Paulo, S. Paulo, Brazil	1
College Misericordia, Dallas, United States	1
Greek Aerospace Medical Association and Space Research, Thessaloniki, Greece	1
Hospital Universitario Río Hortega, Valladolid, Spain	1
Hospital Zamora, Zamora, Spain	1
Oregon Health and Science University, Portland, United States	1
The University of Sheffield International Faculty, Sheffield, Greece	1
University College London, Institute of Mental Health, London, United Kingdom	1
University of Athens Medical School, Athens, Greece	1
University of Florida, Gainesville, United States	1
University of Nottingham, Nottingham, United Kingdom	1
University of Hull, Hull, United Kingdom	1

In the Table 5, we list the information of the ten most cited papers: author, year, title, source, keywords and number of citations (Citats).

**Table 5:** Most cited papers.

Author	Year	Title	Source	Keywords	Citats
Faucounau et al.^36^	2010	Cognitive intervention programmes on patients affected by mild cognitive impairment: A promising intervention tool for MCI?	Journal of Nutrition, Health and Aging. 2010;14(1):31-5	Cognitive stimulation; Cognitive training; Computer-based cognitive intervention; Mild cognitive impairment	50
Viola et al.^38^	2011	Effects of a multidisciplinar cognitive rehabilitation program for patients with mild Alzheimer’s disease	Clinics. 2011;66(8):1395-400	Alzheimer’s disease; Cognition; Quality of life; Rehabilitation; Treatment	44
García-Casal et al.^16^	2017	Computer-based cognitive interventions for people living with dementia: a systematic literature review and meta-analysis	Aging and Mental Health. 2017;21(5):454-67	Alzheimer disease; Cognitive rehabilitation; cognitive stimulation; computer; dementia	33
González-Palau et al.^40^	2014	The effects of a computer-based cognitive and physical training program in a healthy and mildly cognitive impaired aging sample	Aging and Mental Health. 2013;18(7):838-46	Alzheimer’s disease; cognitive stimulation; mild cognitive impairment; mild dementia; physical activity	33
Venneri^57^	2007	Imaging treatment effects in Alzheimer’s disease	Magnetic Resonance Imaging. 2007;25(6):953-68		31
Eckroth-Bucher and Siberski^35^	2009	Preserving cognition through an integrated cognitive stimulation and training program	American Journal of Alzheimer’s Disease and other Dementias. 2009;24(3):234-45	Cognitive impairment; Cognitive stimulation; Cognitive training; Computer-based training	27
Breton et al.^22^	2012	KiMentia: Kinect based tool to help cognitive stimulation for individuals with dementia	2012 IEEE Proceedings of the 14th International Conference on e-Health Networking, Applications and Services (Healthcom). IEEE; 2012. p. 325-8	Dementia; elderly; Kinect; Windows	21
Dodge et al.^24^	2015	Web-enabled conversational interactions as a method to improve cognitive functions: Results of a 6-week randomized controlled trial	Alzheimer’s & Dementia: Translational Research & Clinical Interventions. 2015;1(1):1-12	Communication technology; Conversational interaction; Internet; Mild cognitive impairment; Oregon Center for Aging and Technology (ORCATECH); Prevention study; Randomized controlled clinical trial; Social engagement	21
Westphal et al.^37^	2010	What can low and high technologies do for late-life mental disorders?	Current Opinion in Psychiatry. 2010;23(6):510-5	Aged psychiatry; technology; therapy	14

The country affiliation with the most cited authors is the United States (25%), followed by Spain (21%), Greece (13%), United Kingdom (13%), Brazil (12%), France (8%), Switzerland (4%) and Australia (4%).

We found five keyword clusters (as we can see in Figure 3):

- (C1) Communication technology, conversational interactivity, internet, mild cognitive impairment, Oregon Centre for Aging, prevention study, randomized controlled clinic, and social engagement.
- (C2) Cognitive impairment, cognitive stimulation, cognitive training, computer-based cognitive inter and computer-based training.
- (C3) Alzheimer’s disease, cognition, quality of life, rehabilitation and treatment.
- (C4) Dementia, elderly, Kinect and windows.
- (C5) Alzheimer’s disease, cognitive rehabilitation and therapy.

## DISCUSSION

The purpose of this paper was to analyze the scientific production in CCS and PWD in journals indexed in Clarivate Analytics’ Web of Science and Elsevier’s Scopus since 2000. This work was the first bibliometric review study and very useful about the exploration of the literature CCS and PWD or people with MCI from WoS and Scopus databases, and has outlined the evolutionary trajectory of the collective knowledge over the past 21 years and highlighted the areas of active pursuit.

Let us answer the research questions. The bibliometric questions considered are:

**Q1: When were the articles published?**

The average number of articles is 4 per year. The year with the greatest number of articles is 2018 (10). The curves of publications have an increasing trend until 2018 in both datasets, explaining the progress of scientific literature in this field of research. In 2019, there has been a decrease in published papers.

**Q2: Where were the articles published?**

There are 50 sources of publications: only two journals have three publications (International Journal of Geriatric Psychiatry and Journal of Alzheimer’s Disease) and seven others have two publications (one of these is ACM International Conference Proceeding Series), most of these are from the first quartile. With more than two publications: three journals are from United Kingdom and three from United States.

**Q3: What is the focus of the articles? Has it evolved over the years?**

We found 425 keywords. The 20 most common keyword are “Dementia”, “aged”, “Cognitive stimulation”, “Article”, “Mild cognitive impairment”, “human”, “quality of life”, “Cognition”, “controlled study”, “Alzheimer disease”, “caregiver”, “cognitive defect”, “cognitive therapy”, “Neurodegenerative diseases”, “adult”, “aging”, “Alzheimer’s disease”, “Cognitive stimulation therapy”, “Cognitive stimulations” and “technology”. We found three clusters:

- (C1) Alzheimer’s disease, cognition, quality of life, technology, and treatment.
- (C2) Cognitive impairment, cognitive stimulation, cognitive training, and computer.
- (C3) Cognitive function, internet, and mild cognitive impairment.

The most frequent keywords until 2009 were “Human”, “Alzheimer Disease”, “Article”, “Dementia”, “Donepezil” and “Humans”; from 2010–2014, were “Dementia”, “Aged”, “Human”, “Cognitive Stimulation”, “Female” and “Humans”; from 2015–2019, were “Dementia”, “Human”, “Aged”, “Cognition”, “Cognitive Stimulation” and “Cognitive Stimulations”.

**Q4: Who publishes on the subject? Where do researchers who are interested in PWD work? What country do they live in?**

There are 320 authors. Only three of these 320 wrote three articles: Dodge, Hiroko Hayama (University of Michigan, Ann Arbor, United States), Lenoir, Hermine (Université Paris Descartes, Paris, France) and Meza-Kubo, Victoria (Universidad Autónoma de Baja California, Ensenada, Mexico). There are authors from 20 countries. The countries with the largest number of articles are: United Kingdom (15%), Spain (12%) and United States (10%).

The large projected increase in the number of people with dementia makes finding a treatment to slow or stop dementia as soon as possible essential. The investigations try to find solutions regarding how to deal with dementia in countries with many PWD. As the number of AD cases rises rapidly in an aging global population, the need to understand this puzzling disease is growing and the number of researches is growing in countries like the United Kingdom, Spain and United States.

According to Alzheimer Europe,^46^ examining the population data of the United Kingdom, there is an increase in population for the period 2018 and 2025, with a significant increase in the numbers of people aged over 65, and, in particular, the over 85 age range, which more than doubles between this period. It should be noted that PWD will represent 2.67% of the overall population in 2050 compared to 1.56% in 2018. Villarejo-Galende et al.^47^ performed a literature review of the published evidence and they found that in Spain most population studies of patients older than 65 report prevalence rates ranging from 4 to 9%. Prevalence of dementia and AD is higher in women for nearly every age group. AD is the most common cause of dementia (50–70% of all cases). According to the Alzheimer Association,^1^ millions of Americans have Alzheimer’s or other dementias and, as the size and proportion of the U.S. population age 65 and older continue to increase, the number of Americans with Alzheimer’s or other dementias will grow. The Alzheimer Association^1^ considers that this number will escalate rapidly in coming years, as the population of Americans age. Thus, 65 and older is projected to grow from 55 million in 2019 to 88 million by 2050.

**Q5: What are the most cited articles?**

The most cited papers give historical perspective on scientific progress and reveal recognition of scientific advancement. For this classification, we use the numbers presented in the Scopus database. We found that the positions coincided with the WoS database. We observed that the most cited paper was the Faucounau et al.,^36^ who found that, though both traditional and computer-based cognitive intervention programs seem to be effective, the computer-based ones have more advantages. This paper is in line with the review published studies on CCS and PWD published by Djabelkhir et al.^15^ who considered that computerized cognitive training programs may be used as a practical and valuable tool in clinic to improve cognitive status. However, the most cited paper contrasted with another paper by Gates et al.^48^ that evaluated the effects of at least 12 weeks of computerized cognitive training on maintaining or improving cognitive function and preventing dementia in people with MCI. They concluded that currently available evidence did not allow them to determine whether or not computerized cognitive training will prevent clinical dementia or improve or maintain cognitive function in those who already have evidence of cognitive impairment. In an systematic review article, Irazoki et al.^49^ studies of computerized cognitive interventions for PWD and cognitive impairment were included if they clearly described objectives, users and functioning. On the overall, the programs were aimed at people with different clinical conditions, able to create specific treatments and personalized training, optimized for portable devices, and user-friendly. These authors found that the selected programs differed from each other in terms of objectives, usage mode and characteristics, even if they were used for the same purposes, and they concluded that more information about the features and context of use was needed, as well as more clinical studies, to be able to compare among computerized cognitive programs. This review work was of great importance because the information obtained in the review may be relevant to distinguish programs and select the one that best suits each user.

Some of the most productive institutions involved in CCS and PWD are Department and Institute of Psychiatry, Universidade de São Paulo, São Paulo, Brazil and Université Paris Descartes, Paris, France. The country with the most cited authors is the United States, followed by Spain. The biggest keyword cluster from the most cited articles is communication technology, conversational interactivity, internet, mild cognitive impairment, Oregon Centre for Aging, prevention study, randomized controlled clinic and social engagement.

The progressive increase in the number of scientific papers in CCS and PWD until 2018 likely to combine the effects of a number of factors: the aging of the population,^50^ ^,^ ^51^ the risk of dementia grows exponentially with age,^1^ ^,^ ^2^ ^,^ ^52^ an increase in the global prevalence of dementia;^46^ ^,^ 53 ^,^ ^54^ an increase in awareness of dementia as a serious public health problem;^55^ ^,^ ^56^ non-pharmacological interventions in combination with pharmacotherapies have been considered as the best approach in management of PWD,^56^ and the computer use in its different types has proven to be an ally to those who want to detect and mitigate this disease.^15^

We consider that determining the effectiveness of non-pharmacologic therapies can be difficult because of the large number of existing therapies (including CCS and PWD), the diversity of therapeutic aims, the diverse dementia stages, the diverse types of dementia and the lack of a standard method for carrying out any non-pharmacological therapy.

The search terms identified 61 papers related to the use of computers applied to cognitive stimulation and PWD or people with MCI. We found that there was an increasing trend in the paper publication in CCS and PWD until 2018 in WoS and Scopus, explaining the progress of scientific literature in this field of research. In the last year, we noted that there has been a decrease in published papers. The International Journal of Geriatric Psychiatry and Journal of Alzheimer’s Disease had the largest number of publications in CCS and PWD between 1999 and 2000. Major research’ countries are United Kingdom, Spain and United States, and Aristotle University of Thessaloniki (Greece) is the affiliation author with most cited articles. The most productive organizations in the number of the articles in CCS and PWD for the all period (1999–2020) were the Consiglio Nazionale delle Ricerche. Dodge, Lenoir and Meza-Kubo published the largest number of papers. The most cited paper was Faucounau et al.^36^ In this paper, the authors clearly emphasized the advantages of using the computer-based cognitive intervention, which have more advantages compared to traditional cognitive intervention: intervention tailored to the patient’s neuropsychological pattern and needs; to make an immediate objective comparison with data collected earlier, and thus help in setting up a systematic training.

The 20 most common keyword were “Dementia”, “aged”, “Cognitive stimulation”, “Article”, “Mild cognitive impairment”, “human”, “quality of life”, “Cognition”, “controlled study”, “Alzheimer disease”, “caregiver”, “cognitive defect”, “cognitive therapy”, “Neurodegenerative diseases”, “adult”, “aging”, “Alzheimer’s disease”, “Cognitive stimulation therapy”, “Cognitive stimulations” and “technology’. The limitations of this study are related with the inclusion of studies in English only, introducing language bias.

The findings in these papers were analyzed to find recommendations for future work in this area. We concluded that the CCS has been increasingly used as an intervention tool for PWD and MCI and there still seems to be a possibility for evolution in good quality publications. Further research is needed on CCS for PWD using a standard method for carrying out non-pharmacological intervention.
